# Neural Network-Based Prediction Model to Investigate the Influence of Temperature and Moisture on Vibration Characteristics of Skew Laminated Composite Sandwich Plates

**DOI:** 10.3390/ma14123170

**Published:** 2021-06-09

**Authors:** Vinayak Kallannavar, Subhaschandra Kattimani, Manzoore Elahi M. Soudagar, M. A. Mujtaba, Saad Alshahrani, Muhammad Imran

**Affiliations:** 1 Department of Mechanical Engineering, National Institute of Technology Karnataka, Surathkal 575025, India; subhaskatti@nitk.edu.in; 2 Department of Mechanical Engineering, Glocal University, Delhi-Yamunotri Marg, Uttar Pradesh 247121, India; me.soudagar@gmail.com; 3 Department of Mechanical Engineering, Faculty of Engineering, University of Malaya, Kuala Lumpur 50603, Malaysia; m.mujtaba@uet.edu.pk; 4 Department of Mechanical Engineering, King Khalid University, Guraiger, Abha 62529, Saudi Arabia; 5 Department of Mechanical, Biomedical and Design Engineering, College of Engineering and Physical Sciences, Aston University, Birmingham B4 7ET, UK; m.imran12@aston.ac.uk

**Keywords:** artificial neural network, finite element analysis, shear deformation theory, skew angle, sandwich plates, effect of temperature and moisture

## Abstract

The present study deals with the development of a prediction model to investigate the impact of temperature and moisture on the vibration response of a skew laminated composite sandwich (LCS) plate using the artificial neural network (ANN) technique. Firstly, a finite element model is generated to incorporate the hygro-elastic and thermo-elastic characteristics of the LCS plate using first-order shear deformation theory (FSDT). Graphite-epoxy composite laminates are used as the face sheets, and DYAD606 viscoelastic material is used as the core material. Non-linear strain-displacement relations are used to generate the initial stiffness matrix in order to represent the stiffness generated from the uniformly varying temperature and moisture concentrations. The mechanical stiffness matrix is derived using linear strain-displacement associations. Then the results obtained from the numerical model are used to train the ANN. About 11,520 data points were collected from the numerical analysis and were used to train the network using the Levenberg–Marquardt algorithm. The developed ANN model is used to study the influence of various process parameters on the frequency response of the system, and the outcomes are compared with the results obtained from the numerical model. Several numerical examples are presented and conferred to comprehend the influence of temperature and moisture on the LCS plates.

## 1. Introduction

Polymer composite materials have gained substantial importance in high-end structural engineering fields such as the aerospace and automobile industries [1], biomedical industries [2], construction industries [3], naval industries [4], etc. These structures are often subjected to transverse or bending loads in real-time applications, which impart maximum stresses at the surface of the structure, whereas the center of the structure experiences minimum stresses. Sandwich structures are highly suited for such applications as they are made of a soft and thick core and strong and thin face sheets [5]. These constituents are expected to operate in challenging working atmospheres, such as high operating temperature [6], presence of moisture [7], and electric and magnetic fields [8], and can be incorporated into prominent fields of structural engineering.

Constantly changing environmental conditions may unfavorably affect the performance of materials, which in turn may cause untimely damage to structures. To avoid this, systematic health monitoring mechanisms should be devised. Constant health monitoring of structures enables engineers to detect anomalies in time and begin suitable repair and maintenance work. Additionally, a systematic scientific study may lead to the detection of the source of structural errors, and suitable actions can be planned to avoid future occurrences. The process of employing a damage identification strategy for civil, mechanical, and aerospace engineering structures is generally referred to as structural health monitoring (SHM) [9]. Vibration-based SHM techniques are the predominantly used non-destructive damage detection methods [4,10,11]. High-precision sensors such as accelerometers, strain gauges, velocity transducers, laser displacement sensors, etc., are strategically placed on the structures to obtain the structural responses, mode shapes, natural frequency, damping parameters, etc. Meticulous engineering evaluation of measured responses can detect damage and/or predict the magnitude of damage.

In recent years, substantial effort has been dedicated to understanding the vibrational characteristics of laminated composite structures in the presence of various environmental conditions. Several scholars have investigated the dynamic response of the composite structures such as beams [3,12,13,14], shells [6,7,15,16], and plates [2,17,18] using various analytical, numerical, and experimental methods. Sobhy [12] proposed a new four-variable shear deformation theory to investigate the vibration and buckling behavior of functionally graded (FG) sandwich plates. The studies were performed on the FG sandwich plate resting on a Winkler–Pasternak elastic foundation operating in the hygrothermal environment. Mehar et al. [13] employed higher-order kinematic model isoparametric finite element (FE) steps to investigate the vibration characteristics of multi-walled carbon nanotube-reinforced composite sandwich plate operating in an elevated thermal environment. Obtained numerical simulations are compared with the experimental results. It was reported that the structural stiffness considerably reduced with an increase in temperature, which, in turn, decreases the natural frequency of the structure. Dewangan et al. [14] explored the influence of cutout parameters on the frequency response of the composite plates using numerical and experimental techniques. Cutout parameters such as the shape of cutout, position, and orientation were investigated using a glass/epoxy composite plate.

Recently Katariya et al. [15] numerically and experimentally investigated the vibrational behavior of epoxy-filled softcore skewed laminated composite sandwich (LCS) plates. Numerical analyses were performed using the single-layer higher-order theory, including through-thickness stretching. Numerical results were then compared with the experimental outcomes for various input parameters such as stacking sequence, number of face sheet layers, aspect ratios, etc. Biswal et al. [6,16] performed numerical and experimental investigations to understand the modal behavior of laminated composite shallow shells operating in elevated thermal environments. It was reported that instability occurs at lower excitation frequencies in the presence of a hygrothermal environment. Garg et al. [19] used the improved trigonometric zigzag theory to appreciate the effect of hygro-thermo-mechanical loading on the static behavior of composite and sandwich plates. Several simulations were performed to understand the influence of the length to thickness ratio, loading profile (uniformly distributed and sinusoidal), modular ratio, boundary conditions, thermal and moisture coefficients, etc., on the central deflection of the structure. Chandra et al. [20] performed a stochastic dynamic response investigation of composite plates using generalized polynomial chaos expansion for random temperature increments. Rath and Sahu [21] conducted a numerical and experimental investigation of the impact of the hygrothermal environment on the vibration response of composite plates. The reduction in natural frequency was reported with an increase in temperature and moisture concentration values. Sit and Ray [22] investigated the effect of the hygrothermal environment on the free vibration characteristics of the laminated composite plates made of glass and bamboo fiber mats. Both numerical and experimental results indicated that the reduction percentage in natural frequency values for the bamboo composite plate is higher than the glass epoxy composite plate for all the values of temperature and moisture concentrations considered.

Padhi and Pandit [23] investigated the static and free vibration characteristics of the LCS plate using the refined higher-order zigzag laminate theory. Particular emphasis was focused on low-density core sandwich plates by generating an effective and efficient C^0^ FE formulation. Zenkour and Alghanmi [8] performed a static analysis of sandwich plates made of piezoelectric face sheets and a functionally graded core. The central deflection and the stresses generated in the sandwich plates acted upon by sinusoidal thermo-electro-mechanical loads were reported. Daikh et al. [24] utilized the higher-order shear deformation theory (HSDT) to investigate the static behavior of sandwich plates experiencing the thermo-mechanical loads. Temperature-dependent material properties were considered for the study. Ding et al. [25,26] experimentally investigated the effect of various harsh environmental aging on mechanical characteristics of the sandwich composites. Sandwich composites made of a PVC foam core and vinyl-ester-based composite face sheets were subject to salt-fog spray aging, hygrothermal aging, and solar radiation in combination with water vapor environmental aging.

The developed mathematical model representing the structure under different loading conditions can easily be formulated in the form of computer codes. The generated computer codes can then be used to optimally solve the structural problems by varying process parameters such as the length to thickness ratio, length to width ratio, fiber orientation of the composite structure, etc. These computer codes require a substantial amount of time to solve the problems and generate the results. The accurate predictive models based on the pre-existing dataset are a more appropriate option for SHM applications. The artificial neural network (ANN) techniques are often used for developing predictive models based on the preliminary dataset [27]. The ANN technique has been extensively and effectively employed in civil engineering applications and is slowly gaining importance in other prominent engineering areas as well. In the recent past, ANNs have been used extensively by researchers for structural engineering applications such as failure prediction, crack detection, delamination identification and quantification of magnitude, predicting the size and position of cutouts [28,29], mechanical characteristics [30,31], etc. Recently, Zenzen et al. [32] adopted a transmissibility damage indicator and an ANN to predict damage location and size. The transmissibility function and mode shapes were used to estimate the damage location and then the trained ANN model was used to predict the extent of the damage. The proposed model was intended for fast prediction with highly accurate results without the need to collect all modal analysis data. Gomes et al. [33] used a combination of a genetic algorithm (GA) and an ANN for delamination identification of plate-like structures. Fisher information matrix criteria were used to optimize the sensor position and a feed-forward ANN was used to detect the damage based on data obtained from FE analysis.

Rjoub and Alshatnawi [34] established an analytical mathematical model to investigate the vibration response of simply-supported porous FG plates using Reddy’s third-order plate theory. The results obtained were then used to train the ANN for the natural frequency prediction of the system. It was claimed that the developed ANN model could be easily modified to predict the frequency response for other boundary conditions. Atilla et al. [28] investigated the influence of the location, diameter, and number of circular cutouts on the modal and buckling behavior of composite plates using numerical methods. The results were validated with the experimental results. The ANN model was developed using the Levenberg–Marquardt backpropagation algorithm to predict the natural frequency and buckling loads of composite plates. Elshafey et al. [29] developed an effective ANN model for crack width prediction of thick and thin concrete members using the feed-forward backpropagation method. It was reported that the predicted average crack width results were more accurate than the results obtained using the rules in existing building codes.

Oliver et al. [35] successfully developed an ANN-based damage detection module for composite plates using frequency shifts. The developed damage detection module used the modal data obtained from the FE analysis for training. The severity and position of predicted damage were reported to have 95% accuracy. Jalal et al. [36] developed an optimum ANN model to predict the strength of a rubberized cement composite based on the experimentally obtained characteristics of the composite. The predicted strength values were reported to have 98% accuracy. Jodaei et al. [37] used a state-space-based differential quadrature technique to study the frequency response of functionally graded annular plates. In addition, an optimal ANN model was proposed for the prediction of the natural frequency of the system operating under different boundary conditions. It was reported that the ANN model predicts frequencies with high accuracy and agrees well with the semi-analytical result trend.

From the extensive literature survey, to the best of the authors’ knowledge, work relating to the influence of hygro-thermally induced pre-stresses on the modal behavior of sandwich composites is scarce. In this paper, a methodology is proposed to generate a predictive model to understand the influence of thermal and moisture environments on the free vibration characteristics of skew LCS plates. The methodology adopted is based on the FSDT numerical model and ANN. An ANN prediction model is developed using the numerical data obtained from the validated numerical model. The influence of process parameters such as the effect of the length to breadth (a/b) ratio, length to thickness (a/H) ratio, core thickness to thickness of face sheet (t_c_/t_f_) ratio, fiber orientation, skew angle, and boundary constraints on the vibrational characteristics are investigated under various hygrothermal conditions.

## 2. Mathematical Model

**Figure 1:**
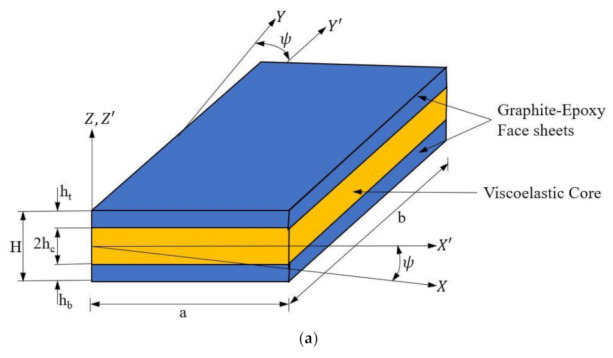

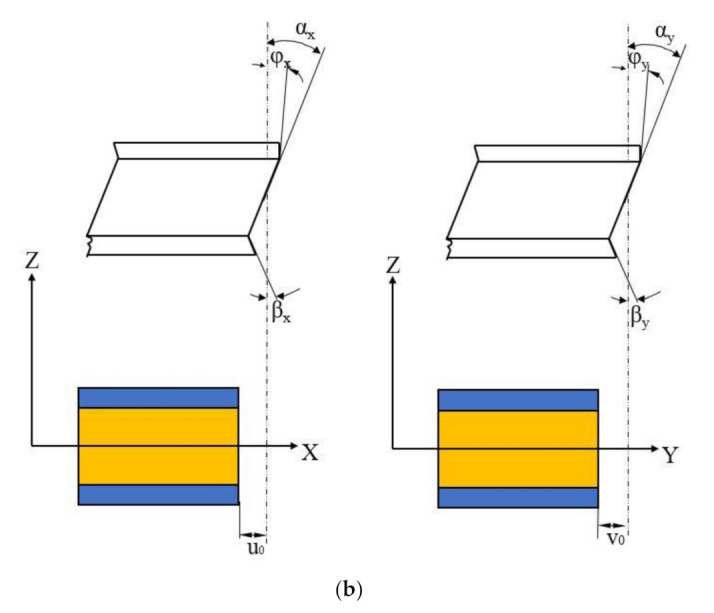
Graphical illustration of the (**a**) Laminated Composite Sandwich (LCS) plate and (**b**) kinematics of deformation of the LCS plate.

Figure 1a demonstrates the schematic illustration of the skew LCS plate. The graphite-epoxy composite laminates are used as the top and bottom face sheets, and DYAD 606 viscoelastic material is used as the core material. The length of the plate is denoted as **a**, and the width and thickness are symbolized as **b** and **H**, respectively. The face sheet thickness is denoted as **h** (**h_t_** *=* **h_b_** *=* **h**), and the core thickness is *2h_c_*. Figure 1b exemplifies the kinematics of the deformation of an LCS plate in XZ- and YZ-planes. The angles **α_x_**, **β_x_**, **φ_x_** and **α_y_**, **β_y_**, **φ_y_**, signify the rotation in XZ- plane and YZ- planes, respectively. At midplane, the translational displacements along X, Y, and Z directions are denoted as x_0_, y_0_, and z_0_, respectively.

### 2.1. Linear Strain Displacement Relations

For easier problem solving, the translational displacements and the rotational variables used separately are as follows:

(1) $$\left\{d_{tra}\right\}={\left[\begin{array}{ccc} x_{0} & y_{0} & z_{0} \end{array}\right]}^{T},\;\mathrm{and}\;\left\{d_{rot}\right\}={\left[\begin{array}{ccccc} \alpha_{x} & \alpha_{y} & \beta_{x} & \beta_{y} & \begin{array}{cc} \varphi_{x} & \varphi_{y} \end{array} \end{array}\right]}^{T}$$

The selective integration law is implemented by considering the strain vectors $\left\{\varepsilon_{b}\right\}$ and $\left\{\varepsilon_{s}\right\}$ as the state of strain at any point in the overall plate. The strain vectors denoting the kinematics of deformation can be expressed as follows [1]:

(2) $$\left\{\varepsilon_{b}\right\}={\left\{\begin{array}{ccc} \varepsilon_{x} & \varepsilon_{y} & \varepsilon_{xy} \end{array}\right\}}^{T},\;\mathrm{and}\;\left\{\varepsilon_{s}\right\}={\left\{\begin{array}{cc} \varepsilon_{xz} & \varepsilon_{yz} \end{array}\right\}}^{T}$$

where:

ε_x_, ε_y_: Strains along x and y directions.
ε_xy_: In-plane shear strain.
ε_xz_, ε_yz_: Transverse shear strains.

The strain vectors ${\left\{\varepsilon_{b}\right\}}_{core}$, ${\left\{\varepsilon_{b}\right\}}_{bot}$, and ${\left\{\varepsilon_{b}\right\}}_{top}$ defining the state of normal strains and in-plane transverse shear strain at any point in the core, and the bottom and top face sheets can be expressed as

(3) $${\left\{\varepsilon_{b}\right\}}_{core}=\left\{\begin{array}{c} \frac{\partial x_{0}}{\partial x}+z\frac{\partial \alpha_{x}}{\partial x}\\ \frac{\partial y_{0}}{\partial y}+z\frac{\partial \alpha_{y}}{\partial y}\\ \left(\frac{\partial x_{0}}{\partial y}+\frac{\partial y_{0}}{\partial x}\right)+z\left(\frac{\partial \alpha_{x}}{\partial y}+\frac{\partial \alpha_{y}}{\partial x}\right) \end{array}\right\}$$

(4) $${\left\{\varepsilon_{b}\right\}}_{bot}=\left\{\begin{array}{c} \frac{\partial x_{0}}{\partial x}-h_{c}\frac{\partial \alpha_{x}}{\partial x}+(z+h_{c})\frac{\partial \beta_{x}}{\partial x}\\ \frac{\partial y_{0}}{\partial y}-h_{c}\frac{\partial \alpha_{y}}{\partial y}+(z+h_{c})\frac{\partial \beta_{y}}{\partial y}\\ \left(\frac{\partial x_{0}}{\partial y}+\frac{\partial y_{0}}{\partial x}\right)-h_{c}\left(\frac{\partial \alpha_{x}}{\partial y}+\frac{\partial \alpha_{y}}{\partial x}\right)+(z+h_{c})\left(\frac{\partial \beta_{x}}{\partial y}+\frac{\partial \beta_{y}}{\partial x}\right) \end{array}\right\}$$

(5) $${\left\{\varepsilon_{b}\right\}}_{top}=\left\{\begin{array}{c} \frac{\partial x_{0}}{\partial x}+h_{c}\frac{\partial \alpha_{x}}{\partial x}+(z-h_{c})\frac{\partial \varphi_{x}}{\partial x}\\ \frac{\partial y_{0}}{\partial y}+h_{c}\frac{\partial \alpha_{y}}{\partial y}+(z-h_{c})\frac{\partial \varphi_{y}}{\partial y}\\ \left(\frac{\partial x_{0}}{\partial y}+\frac{\partial y_{0}}{\partial x}\right)+h_{c}\left(\frac{\partial \alpha_{x}}{\partial y}+\frac{\partial \alpha_{y}}{\partial x}\right)+(z-h_{c})\left(\frac{\partial \varphi_{x}}{\partial y}+\frac{\partial \varphi_{y}}{\partial x}\right) \end{array}\right\}$$

On simplification and rearranging the terms, the strains can be expressed as

(6) $$\begin{array}{l} {\left\{\varepsilon_{b}\right\}}_{core}=\left\{\varepsilon_{bt}\}+\left[Z_{1}\right]\{\varepsilon_{br}\right\}\\ {\left\{\varepsilon_{b}\right\}}_{bot}=\left\{\varepsilon_{bt}\}+\left[Z_{2}\right]\{\varepsilon_{br}\right\}\\ {\left\{\varepsilon_{b}\right\}}_{top}=\left\{\varepsilon_{bt}\}+\left[Z_{3}\right]\{\varepsilon_{br}\right\} \end{array}$$

Similarly, the strain vectors ${\left\{\varepsilon_{s}\right\}}_{core}$, ${\left\{\varepsilon_{s}\right\}}_{bot}$, and ${\left\{\varepsilon_{s}\right\}}_{top}$ define the transverse shear strains at any location in the structure:

(7) $${\left\{\varepsilon_{s}\right\}}_{core}=\left\{\begin{array}{c} \frac{\partial z_{0}}{\partial x}+\alpha_{x}\\ \frac{\partial z_{0}}{\partial y}+\alpha_{y} \end{array}\right\};\;{\left\{\varepsilon_{s}\right\}}_{bot}=\left\{\begin{array}{c} \frac{\partial z_{0}}{\partial x}+\beta_{x}\\ \frac{\partial z_{0}}{\partial y}+\beta_{y} \end{array}\right\};\;{\left\{\varepsilon_{s}\right\}}_{top}=\left\{\begin{array}{c} \frac{\partial z_{0}}{\partial x}+\varphi_{x}\\ \frac{\partial z_{0}}{\partial y}+\varphi_{y} \end{array}\right\}$$

On simplification and rearranging, we have

(8) $$\begin{array}{l} {\left\{\varepsilon_{s}\right\}}_{core}=\left\{\varepsilon_{st}\}+\left[Z_{4}\right]\{\varepsilon_{sr}\right\}\\ {\left\{\varepsilon_{s}\right\}}_{bot}=\left\{\varepsilon_{st}\}+\left[Z_{5}\right]\{\varepsilon_{sr}\right\}\\ {\left\{\varepsilon_{s}\right\}}_{top}=\left\{\varepsilon_{st}\}+\left[Z_{6}\right]\{\varepsilon_{sr}\right\} \end{array}$$

The various matrices presented in Equations (6) and (8) are elaborated in Appendix A. The complex modulus approach is used for demonstrating the viscoelastic material. The storage modulus and loss factor are considered temperature-dependent, and hence the shear modulus of the viscoelastic material can be denoted as

(9) $$G(T)=G^{\prime }(T)[1+i\eta (T)]$$

in which, $G^{\prime }$ and η are the storage modulus and the loss factors, respectively.

### 2.2. Non-Linear Strain Displacement Relations

The non-linear strains of the plate can be articulated as [21]

(10) $$\begin{array}{l} \varepsilon_{xnl}=\frac{1}{2}[x_{0}{}_{,x}^{2}+y_{0}{}_{,x}^{2}+z_{0}{}_{,x}^{2}+2z(x_{0}{}_{,x}\theta_{y,x}-y_{0}{}_{,x}\theta_{x,x})+z^{2}(\theta_{y,x}^{2}+\theta_{x,x}^{2})]\\ \varepsilon_{ynl}=\frac{1}{2}[x_{0}{}_{,y}^{2}+y_{0}{}_{,y}^{2}+z_{0}{}_{,y}^{2}+2z(x_{0}{}_{,y}\theta_{y,y}-y_{0}{}_{,y}\theta_{x,y})+z^{2}(\theta_{y,y}^{2}+\theta_{x,y}^{2})]\\ \varepsilon_{xynl}=[x_{0}{}_{,x}x_{0}{}_{,y}+y_{0}{}_{,x}y_{0}{}_{,y}+z_{0}{}_{,x}z_{0}{}_{,y}+z(x_{0}{}_{,x}\theta_{y,y}+x_{0}{}_{,y}\theta_{y,x}-y_{0}{}_{,x}\theta_{x,y}-y_{0}{}_{,y}\theta_{x,x})+z^{2}(\theta_{y,x}\theta_{y,y}+\theta_{x,x}\theta_{x,y})]\\ \varepsilon_{xznl}=[x_{0}{}_{,x}\theta_{y}-y_{0}{}_{,x}\theta_{x}+z(\theta_{y}\theta_{y,x}+\theta_{x}\theta_{x,x})]\\ \varepsilon_{yznl}=[x_{0}{}_{,y}\theta_{y}-y_{0}{}_{,y}\theta_{x}+z(\theta_{y}\theta_{y,y}+\theta_{x}\theta_{x,y})] \end{array}$$

where x_0,x_ indicates a partial derivative of *x*_0_ with respect to x, i.e., x0,x=∂x0∂x.

### 2.3. Finite Element Model

The eight-noded isoparametric quadrilateral elements are used to mesh or discretize the complete plate. Three translational (x_0_, y_0_, z_0_) and six rotational (α_x_, α_y_, β_x_, β_y_, φ_x_, φ_y_) degrees of freedom are considered at each node. In general, the displacement vectors of any element can be articulated as

(11) $$\left\{d_{trai}\right\}={\left[\begin{array}{ccc} x_{0i} & y_{0i} & z_{0i} \end{array}\right]}^{T},\;\mathrm{and}\;\;\left\{d_{roti}\right\}={\left[\begin{array}{ccccc} \alpha_{xi} & \alpha_{yi} & \beta_{xi} & \beta_{yi} & \begin{array}{cc} \varphi_{xi} & \varphi_{yi} \end{array} \end{array}\right]}^{T}$$

Thus, the elemental generalized displacement vectors can be formed using the generalized nodal vectors:

(12) $$\left\{d_{tra}\right\}=[N_{tra}]\{d_{tra}^{e}\},\;\mathrm{and}\;\left\{d_{rot}\right\}=[N_{rot}]\{d_{rot}^{e}\}$$

in which,

$$\begin{array}{c} {}[N_{tra}]={\left[N_{tra1}\;N_{tra2}\;\ldots \;N_{tra8}\right]}^{T},\;\mathrm{and}\;[N_{rot}]={\left[N_{rot1}\;N_{rot2}\;\ldots \;N_{rot8}\right]}^{T}\\ N_{trai}=n_{i}I_{tra},\;N_{roti}=n_{i}I_{rot}\\ \{d_{tra}^{e}\}={\left[{\left\{d_{tra1}^{e}\right\}}^{T}\;{\left\{d_{tra2}^{e}\right\}}^{T}\;\ldots \;{\left\{d_{tra8}^{e}\right\}}^{T}\right]}^{T}\;\mathrm{and}\\ \{d_{rot}^{e}\}={\left[{\left\{d_{rot1}^{e}\right\}}^{T}\;{\left\{d_{rot2}^{e}\right\}}^{T}\;\ldots \;{\left\{d_{rot8}^{e}\right\}}^{T}\right]}^{T} \end{array}$$

where I_tra_ and I_rot_ are (3 × 3) and (6 × 6) identity matrices, respectively, and n_i_ is the shape function of the natural coordinate associated with the i-th node.

### 2.4. Elemental Stiffness Matrix

The well-known strain displacement relation can be expressed as

(13) $$\left\{\varepsilon \}=[B]\{\delta_{e}\right\}$$

where

$$\{\delta_{e}\}={\left\{x_{01},y_{01},z_{01},\alpha_{x1},\alpha_{y1},\beta_{x1},\beta_{y1},\varphi_{x1},\varphi_{x2},\ldots ,x_{08},y_{08},z_{08},\alpha_{x8},\alpha_{y8},\beta_{x8},\beta_{y8},\varphi_{x8},\varphi_{y8}\right\}}^{T}$$

The elemental strain vectors at any arbitrary point can be articulated as

(14) $$\begin{array}{l} {\left\{\varepsilon_{b}\right\}}_{core}=[B_{tb}]\{d_{t}^{e}\}+[Z_{1}][B_{rb}]\{d_{r}^{e}\}\\ {\left\{\varepsilon_{b}\right\}}_{bot}=[B_{tb}]\{d_{t}^{e}\}+[Z_{2}][B_{rb}]\{d_{r}^{e}\}\\ {\left\{\varepsilon_{b}\right\}}_{top}=[B_{tb}]\{d_{t}^{e}\}+[Z_{3}][B_{rb}]\{d_{r}^{e}\},\\ {\left\{\varepsilon_{s}\right\}}_{core}=[B_{ts}]\{d_{t}^{e}\}+[Z_{4}][B_{rs}]\{d_{r}^{e}\}\\ {\left\{\varepsilon_{s}\right\}}_{bot}=[B_{ts}]\{d_{t}^{e}\}+[Z_{5}][B_{rs}]\{d_{r}^{e}\}\\ {\left\{\varepsilon_{s}\right\}}_{top}=[B_{ts}]\{d_{t}^{e}\}+[Z_{6}][B_{rs}]\{d_{r}^{e}\} \end{array}$$

The terms [B*_tb_*], [B*_rb_*], [B*_ts_*] and [B*_rs_*] are elaborated in Appendix B.

The dynamic version of the principle of virtual work is incorporated to obtain the equations of motion. The potential and kinetic energy of the structure can be expressed as

(15) $$T_{p}^{e}=\frac{1}{2}\left[{\left\{d_{tra}^{e}\right\}}^{T}\left[K_{11}^{e}\right]\left\{d_{tra}^{e}\right\}+{\left\{d_{tra}^{e}\right\}}^{T}\left[K_{12}^{e}\right]\left\{d_{rot}^{e}\right\}+{\left\{d_{rot}^{e}\right\}}^{T}{\left[K_{12}^{e}\right]}^{T}\left\{d_{tra}^{e}\right\}+{\left\{d_{rot}^{e}\right\}}^{T}\left[K_{22}^{e}\right]\left\{d_{rot}^{e}\right\}-2{\left\{d_{tra}^{e}\right\}}^{T}\left\{F^{e}\right\}\right]$$

(16) $$T_{k}^{e}=\frac{1}{2}\int_{0}^{a_{e}}\int_{0}^{b_{e}}\bar{m}{\left\{{\dot{d}}_{tra}^{e}\right\}}^{T}{\left[N\right]}^{T}\left[N\right]\left\{{\dot{d}}_{tra}^{e}\right\}dxdy$$

The elemental stiffness matrix, the mass matrix, and the force vector appearing in Equations (16) and (17) can be expressed as

(17) $$\left[K_{11}^{e}\right]=\left[K_{11b}^{e}\right]+\left[K_{11s}^{e}\right]$$

(18) $$\left[K_{12}^{e}\right]=\left[K_{12b}^{e}\right]+\left[K_{12s}^{e}\right]$$

(19) $$\left[K_{22}^{e}\right]=\left[K_{22b}^{e}\right]+\left[K_{22s}^{e}\right]$$

(20) $$\left\{F^{e}\right\}=\int_{0}^{a_{e}}\int_{0}^{b_{e}}{\left[N_{tra}\right]}^{T}\left\{f\right\}dxdy$$

(21) $$\bar{m}=\sum_{k=1}^{N}\rho_{bot}\left(h_{k+1}-h_{k}\right)+2\rho_{core}h_{c}+\sum_{k=1}^{N}\rho_{top}\left(h_{k+1}-h_{k}\right)$$

(22) $$\left[M^{e}\right]=\int_{0}^{a_{e}}\int_{0}^{b_{e}}\bar{m}{\left[N_{t}\right]}^{T}\left[N_{t}\right]dxdy$$

The matrices used in Equations (17)–(22) are presented in Appendix C. On solving and simplification, the equation of motion can be expressed as,

(23) $$\left[M^{e}\right]\left\{{\ddot{d}}_{tra}^{e}\right\}+\left[K_{11}^{e}\right]\left\{d_{tra}^{e}\right\}+\left[K_{12}^{e}\right]\left\{d_{rot}^{e}\right\}=\left\{F^{e}\right\}$$

(24) $$\left[M^{e}\right]\left\{{\ddot{d}}_{tra}^{e}\right\}+\left[K_{11}^{e}\right]\left\{d_{tra}^{e}\right\}+\left[K_{12}^{e}\right]\left\{d_{rot}^{e}\right\}=\left\{F^{e}\right\}$$

From the above equations,

(25) $$\left[K_{e}\right]=\left[K_{11}\right]-\left[K_{12}\right]{\left[K_{22}\right]}^{-1}{\left[K_{12}\right]}^{T}$$

### 2.5. Element Initial Stress Stiffness Matrix

The hygrothermal force and moment resultants are stated as

(26) $${\left\{N_{x}^{N},\;N_{y}^{N},\;N_{xy}^{N}\right\}}^{T}=\sum_{k=1}^{n}{\left[{\bar{C}}_{ij}\right]}_{k}{\left\{e\right\}}_{k}(z_{k}-z_{k-1})\;for\;i,\;j=1,\;2,\;6$$

(27) $${\left\{M_{x}^{N},{\;M}_{y}^{N},{\;M}_{xy}^{N}\right\}}^{T}=\frac{1}{2}\sum_{k=1}^{n}{\left[{\bar{C}}_{ij}\right]}_{k}{\left\{e\right\}}_{k}(z_{k}^{2}-z_{k-1}^{2})\;for\;i,\;j=1,\;2,\;6$$

where

(28) $$\begin{array}{l} {\left[{\bar{C}}_{ij}\right]}_{k}={\left[T_{1}\right]}^{-1}{\left[C_{ij}\right]}_{k}\left[T_{1}\right]\;(i,\;j=1,\;2,\;6)\\ {\left[{\bar{C}}_{ij}\right]}_{k}={\left[T_{2}\right]}^{-1}{\left[C_{ij}\right]}_{k}[T_{2}]\;(i,\;j=4,\;5) \end{array}$$

in which,

T_1_ and T_2_ are transformation matrices of order 3 × 3 and 2 × 2 respectively, and,

$${\left[C_{ij}\right]}_{k}=\left[\begin{array}{ccc} C_{11} & C_{12} & 0\\ C_{21} & C_{22} & 0\\ 0 & 0 & C_{66} \end{array}\right]\;(i,j=1,2,6)\;{\left[C_{ij}\right]}_{k}=\left[\begin{array}{cc} C_{44} & 0\\ 0 & C_{55} \end{array}\right]\;(i,j=4,5)$$

where

$$C_{11}=\frac{E_{1}}{\left(1-\upsilon_{12}\upsilon_{21}\right)};\;C_{12}=C_{21}=\frac{\upsilon_{12}E_{2}}{\left(1-\upsilon_{12}\upsilon_{21}\right)};\;C_{22}=\frac{E_{2}}{\left(1-\upsilon_{12}\upsilon_{21}\right)}\;C_{66}=G_{12},\;C_{44}=G_{13},\;C_{55}=G_{23}$$

and

(29) $${\left\{e\right\}}_{k}={\left\{e_{x},{\;e}_{y},{\;e}_{xy}\right\}}^{T}=[\bar{T}]{\left\{\begin{array}{cc} \beta_{1} & \beta_{2} \end{array}\right\}}_{k}^{T}(C-C_{0})+[\bar{T}]{\left\{\begin{array}{cc} \alpha_{1} & \alpha_{2} \end{array}\right\}}_{k}^{T}(T-T_{0})$$

in which,

$$\bar{T}=\left[\begin{array}{cc} \cos^{2}\theta & sin^{2}\theta \\ sin^{2}\theta & \cos^{2}\theta \\ \sin2\theta & \cos2\theta \end{array}\right]$$

where

e_x_, e_y_, e_xy_: Non-mechanical strains
β_1_ and β_2_: Moisture coefficients
α_1_ and α_2_: Thermal coefficients
T and T_0_: Elevated and reference temperature
C and C_0_: Elevated and reference moisture profiles.

The non-linear strains can be expressed as

(30) $$\{\varepsilon_{nl}\}=\{\varepsilon_{xnl},\varepsilon_{ynl},\varepsilon_{xynl}\}=[R]\{d\}/2$$

where

$$\{d\}={\left\{x_{0}{}_{,x},x_{0}{}_{,y},y_{0}{}_{,x},y_{0}{}_{,y},z_{0}{}_{,x},z_{0}{}_{,y},\alpha_{x,x},\alpha_{x,y},\alpha_{y,x},\alpha_{y,y},\beta_{x,x},\beta_{x,y},\beta_{y,x},\beta_{y,y},\varphi_{x,x},\varphi_{x,y},\varphi_{y,x},\varphi_{y,y},\alpha_{x},\alpha_{y},\beta_{x},\beta_{y},\varphi_{x},\varphi_{y}\right\}}^{T}$$

Equation {d} can be expressed as

(31) $$\left\{d\}=[G]\{\partial_{e}\right\}$$

where

$$[G]=\sum_{i=1}^{8}\left[\begin{array}{ccc} \hat{N} & \bar{O} & \bar{O}\\ \bar{O} & \hat{N} & \bar{O}\\ \bar{O} & \bar{O} & \hat{N}\\ \tilde{O} & \hat{I} & \tilde{O}\\ \tilde{O} & \tilde{O} & \hat{I} \end{array}\right],\;\mathrm{in}\;\mathrm{which}\;\hat{N}=\left[\begin{array}{ccc} N_{i,x} & 0 & 0\\ N_{i,y} & 0 & 0\\ 0 & N_{i,x} & 0\\ 0 & N_{i,y} & 0\\ 0 & 0 & N_{i,x}\\ 0 & 0 & N_{i,y} \end{array}\right]$$

$\bar{O}$ and $\tilde{O}$ are (6 × 3) and (3 × 3) null matrices, respectively. Similarly, $\hat{I}$ is the identity matrix of size (3 × 3).

The initial stress stiffness matrix of the non-mechanical loads is given by

(32) $$[K_{\sigma }^{e}]=\int_{-1}^{+1}\int_{-1}^{+1}{\left[G\right]}^{T}[S][G]\left|J\right|d\xi d\eta$$

The terms represented in the above equation are denoted in Appendix D.

### 2.6. Solution Process

Two-point and three-point Gaussian integration rules are utilized to obtain the elemental bending and transverse shear deformation matrices, respectively. The global stiffness matrix [K_e_], initial stress stiffness matrix [K_σ_], and mass matrix [M] are obtained by combining respective elemental matrices [K^e^_e_], [K^e^_σ_], and [M^e^], respectively. The obtained global matrices can be arranged to calculate the natural frequency of the system as

(33) $$\left|\left[\left[K_{e}]+[K_{\sigma }\right]\right]-\omega^{2}[M]\right|=0$$

where ω is the natural frequency of the system. For skew plates, the generalized displacement vectors can be given by

(34) $$\left\{d_{tra}\}=[L_{1}]\{{d^{\prime }}_{tra}\},\;\{d_{rot}\}=[L_{2}]\{{d^{\prime }}_{rot}\right\}$$

where $\left\{{d^{\prime }}_{tra}\right\}$ and $\left\{{d^{\prime }}_{rot}\right\}$ are new generalized displacement vectors

(35) $$\left\{{d^{\prime }}_{tra}\right\}={\left[\begin{array}{ccc} {x^{\prime }}_{0} & {y^{\prime }}_{0} & {z^{\prime }}_{0} \end{array}\right]}^{T},\;\mathrm{and}\;\left\{{d^{\prime }}_{r}\right\}={\left[\begin{array}{ccccc} {\theta^{\prime }}_{x} & {\theta^{\prime }}_{y} & {\phi^{\prime }}_{x} & {\phi^{\prime }}_{y} & \begin{array}{cc} {\alpha^{\prime }}_{x} & {\alpha^{\prime }}_{y} \end{array} \end{array}\right]}^{T}$$

The transformation matrices are given as

(36) $$[L_{1}]=\left[\begin{array}{ccc} \cos\Psi & \sin\Psi & 0\\ -\sin\Psi & \cos\Psi & 0\\ 0 & 0 & 1 \end{array}\right]$$

(37) $$[L_{2}]=\left[\begin{array}{ccc} L_{3} & O_{L} & O_{L}\\ O_{L} & L_{3} & O_{L}\\ O_{L} & O_{L} & L_{3} \end{array}\right];\;\left[L_{3}\right]=\left[\begin{array}{cc} \cos\Psi & \sin\Psi \\ -\sin\Psi & \cos\Psi \end{array}\right]$$

The MATLAB—R2017a simulation tool is used to generate the computer codes for the developed FE model.

## 3. Material Properties

**Figure 2:**
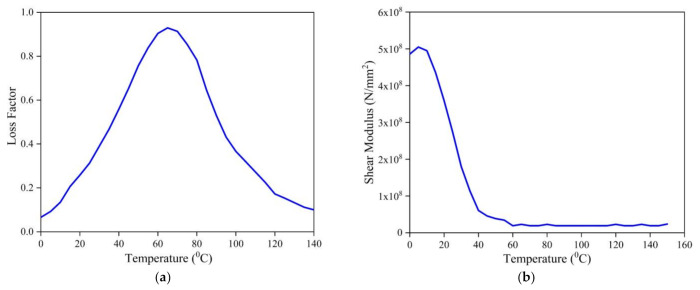
Properties of DYAD 606 viscoelastic material (**a**) loss factor and (**b**) shear modulus variations [10] (Adapted with permission from Elsevier B.V., License Number: 5077631407971).

To appreciate the influence of thermal and moisture environment on the dynamic behavior of the LCS plate, simulations were performed considering graphite–epoxy composite face sheets and DYAD 606 viscoelastic core. The material properties of the constituent material are considered temperature-dependent, as listed in Table 1, Table 2, and Figure 2.

The moisture and temperature-dependent material properties of the graphite–epoxy composite material listed in Table 2a,b are not continuous. Hence, Equations (38) and (39) are generated using curve fitting techniques to interpolate the experimental modulus values listed in Table 2a,b.

Moisture-dependent properties:

(38) $$\begin{array}{l} E_{1}[GPa]=130\\ E_{2}[GPa]=\left(\left(3.588\times 10^{-15}\times M^{2}\right)+M\right)+9.5\\ G_{12}[GPa]=6 \end{array}$$

Temperature-dependent properties:

(39) $$\begin{array}{l} E_{1}[GPa]=130\\ E_{2}[GPa]=\left(5.3333\times 10^{-8}\times \Delta T^{4}\right)-\left(1.3333\times 10^{-5}\times \Delta T^{3}\right)+\left(0.001167\times \Delta T^{2}\right)-(0.06167\times \Delta T)+9.5\\ G_{12}[GPa]=2.168\times e^{-{\left(\frac{\Delta T-23.24}{33.34}\right)}^{2}}+4.844\times e^{-{\left(\frac{\Delta T+84.4}{105.2}\right)}^{2}}+3.007\times e^{-{\left(\frac{\Delta T+16.19}{27.43}\right)}^{2}} \end{array}$$

## 4. Results and Discussions

The mathematical model developed in the preceding segment is used to study the influence of the thermal and moisture environment on the frequency response of the LCS plates. The FE simulations were performed considering uniform temperature and moisture concentration increases.

### 4.1. Comparison with Previous Studies

The non-dimensional frequency for the clamped laminated sandwich plates operating in elevated temperature and moisture conditions are obtained considering a length to thickness (a/H) ratio of 100 and length to width (a/b) ratio of 0.5. The non-dimensional form of the natural frequency is represented as

(40) $$\bar{\omega }=\frac{\omega a^{2}}{\pi^{2}H}\sqrt{\frac{\rho }{E_{t}}}$$

The results obtained for different mesh sizes are listed in Table 3. The outcomes show that the mesh size of 12 × 12 shows excellent convergence with published results [39]. Hence, the mesh dimension of 12 × 12 is used throughout the analysis.

Further, studies are performed on simply supported cross-ply skew sandwich plates considering the a/b ratio as 2 and the a/H ratio as 40. The outcomes attained for skew sandwich composite plates are listed in Table 4. From Table 4, it is obvious that the results obtained in the present formulation are in line with the reference outcomes [40,41].

### 4.2. Artificial Neural Network

An artificial neural network is an information processing computational model made of an interconnected group of artificial neurons [42]. ANNs are capable of learning and processing like a typical human brain, hence the name. ANNs are often used for data fitting and pattern recognition. The trained ANN can then be used to predict or estimate a new independent dataset. The ANN consists of three main parts, namely, the input layer, hidden layer, and output layer. All three layers are interconnected through the synapse to transfer signals or information among each other. Each connection has its weights that change in line with the learning procedure until the expected outcomes are achieved. The hidden layer is composed of a summation unit and an activation block. The summation unit adds the input products and their weights to the biases. Bias provides flexibility for the activation function to efficiently map the input and outputs. The activation function introduces nonlinear properties to the network, which enables the network to generate a relationship for complex nonlinear models. The sigmoidal mathematical function and hyperbolic tangent mathematical functions are commonly used activation functions to solve engineering problems. The training process is repeated to adjust the weights till the expected results are obtained [43].

**Figure 3:**
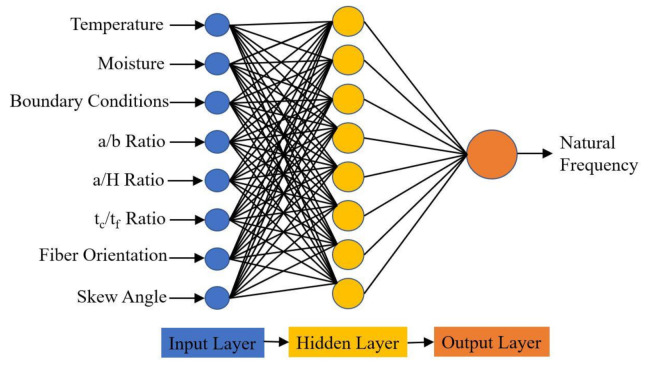
The architecture of the proposed ANN prediction model.

In the present study, 11,520 non-dimensional natural frequency data points were collected from the numerical model developed in the previous section. The MATLAB software package was used to incorporate the feed-forward backpropagation network. A sigmoid transfer function was used for the input and hidden layer, and the linear transfer function was used for the output layer. The parameters considered to generate the dataset are listed in Table 5, and the architecture employed is schematically represented in Figure 3.

**Figure 4:**
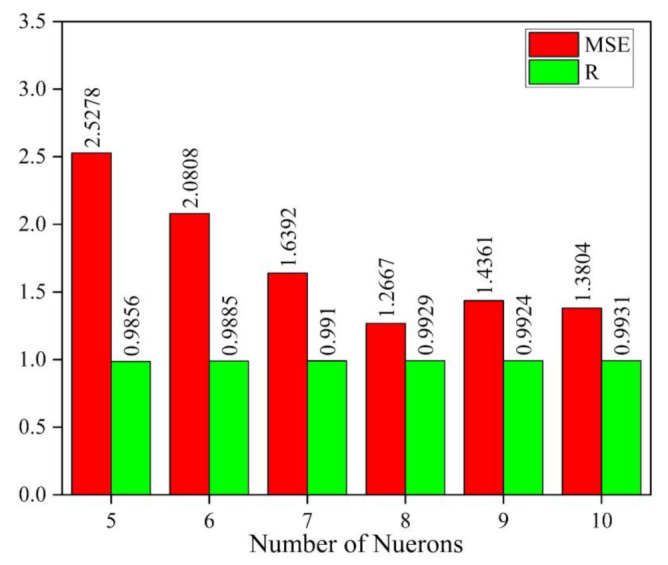
Comparison of MSE and R values for optimal selection of the number of neurons.

The data were divided into 70% for training, 15% for validation, and 15% for testing. The Levenberg–Marquardt algorithm was used to train the network. The trial-and-error method was used to find the optimum number of neurons to model the network architecture with eight inputs and one output. From Figure 4, it is evident that the ANN architecture with a single layer and eight neurons is an optimal option as it has the minimum mean square error (MSE) and maximum correction coefficient (R) value, i.e., 1.2667 and 0.9924, respectively.

**Figure 5:**
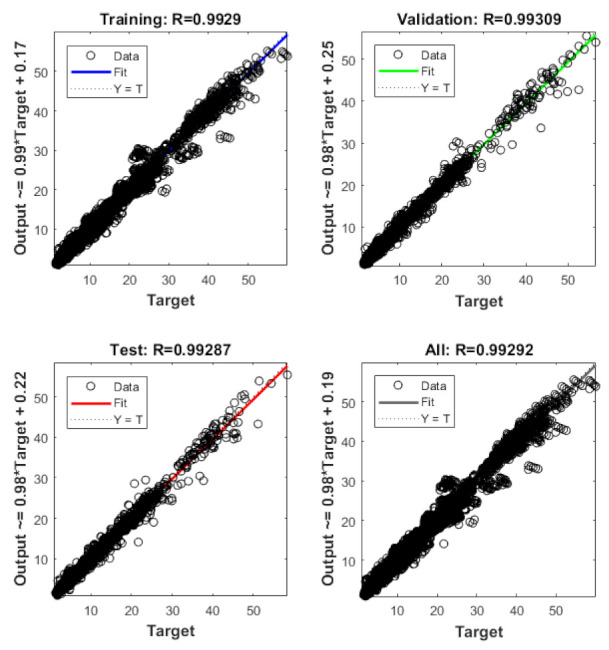
Regression results of training, validation, and test results of neural network.

**Figure 6:**
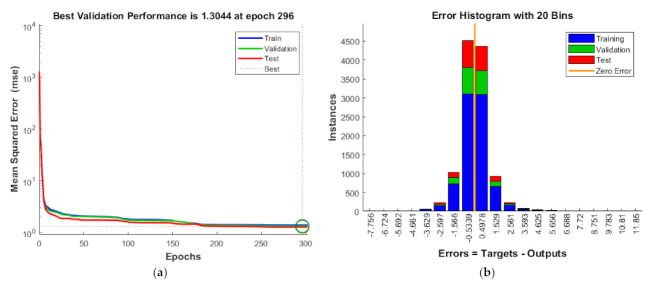
Schematic representation of (**a**) ANN training performance and (**b**) histograms of error values of the developed predictive model.

**Figure 7:**
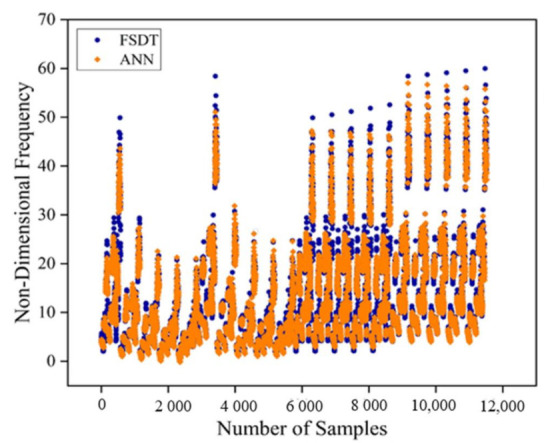
Scatter plot to compare the target results and the ANN predicted fundamental natural frequency values.

The developed ANN architecture was trained using a dataset obtained from the numerical method. From Figure 5, it is evident that the proposed ANN provides a good estimate of the results of the numerical model. The training, validation, and testing show coefficient correlation values as 0.9929, 0.9931, and 0.9929, respectively. The overall coefficient relation value of 0.9929 was observed. The global training results (performance) and the histogram of error of the proposed architecture are presented in Figure 6. To comprehensively evaluate the developed ANN model, the ANN results and the numerical results are plotted in Figure 7. From Figure 7, it can be observed that the predicted results of the ANN have good accuracy. The developed ANN model is then used to predict the influence of the thermal and moisture environment on the vibration response of the LCS plate. Information regarding weight and bias values is shown in Appendix E.

### 4.3. Model Simulation Results

**Figure 8:**
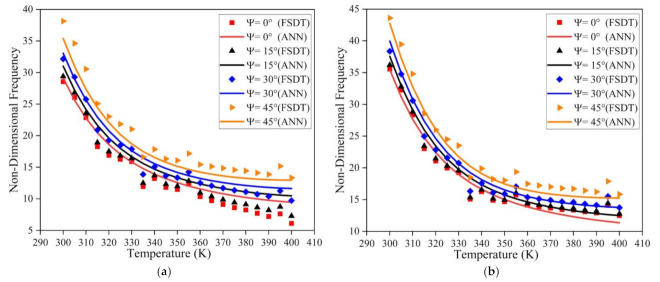
Influence of thermal environment on the fundamental frequency of the LCS plate under (**a**) SSSS and (**b**) CCCC boundary conditions.

The influence of temperature on the frequency response of the LCS plate is investigated considering all sides simply supported (SSSS) and clamped (CCCC) boundary conditions. The length to thickness ratio of 50, length to breadth ratio of 2, and the t_c_/t_f_ ratio of 2 is considered for the simulation. The non-dimension frequency values for varying temperature and skew angle are presented in Figure 8. For both SSSS and CCCC boundary conditions, the natural frequency follows a decreasing trend with increasing temperature values. The frequency value increases with an increase in skew angle for all the temperature values considered. From Figure 8, it is also evident that the results predicted by the ANN model have good accuracy and show a similar trend to the results obtained from the numerical model.

**Figure 9:**
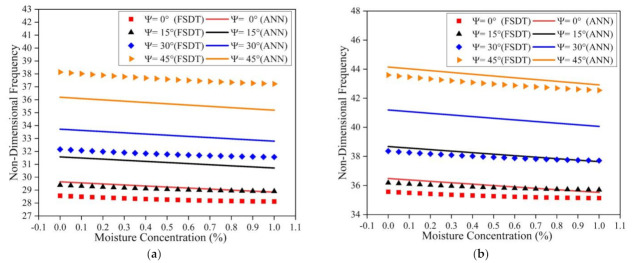
Influence of moisture environment on the fundamental frequency of the LCS plate under (**a**) SSSS and (**b**) CCCC boundary conditions.

Simulations were also carried out to understand the effect of moisture on the natural frequency of the LCS plate operating under SSSS and CCCC boundary constraints. The ANN prediction results and the numerical simulation results are presented in Figure 9. From the results, it is clear that the frequency of the structure decreases with an upsurge in moisture concentration values for both of the boundary conditions considered. For all the moisture concentration values considered, the natural frequency values showed an increasing trend with an increase in the skew angle. It can also be observed that the results predicted by the ANN model have good accuracy. From Figure 8 and Figure 9, it is evident that the variation in the natural frequency predicted by the ANN model is in line with the trend followed by the numerical model in the presence of thermal and moisture environments.

The material properties of composite materials generally degrade when they are exposed to elevated temperature and moisture environments. This, in turn, decreases the stiffness of the composite structure, i.e., with an increase in temperature and moisture, the stiffness of the composite structure decreases. As the stiffness of any system is directly proportional to the natural frequency of that system, the natural frequency of the structure decreases with an increase in temperature and moisture concentration values [20,21,38].

**Figure 10:**
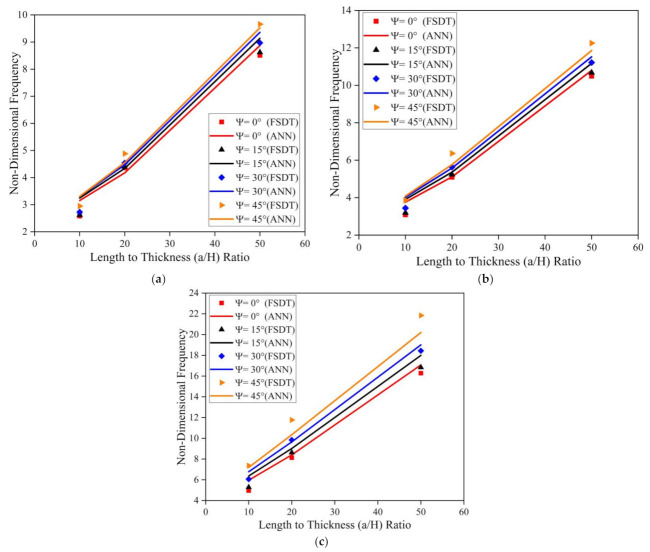
Influence of skew angle on the simply supported skew LCS plate operating at elevated thermal environment (325 K) for varying a/b and a/H ratios (**a**) a/b = 0.5 (**b**) a/b = 1.0 (**c**) a/b = 2.0.

**Figure 11:**
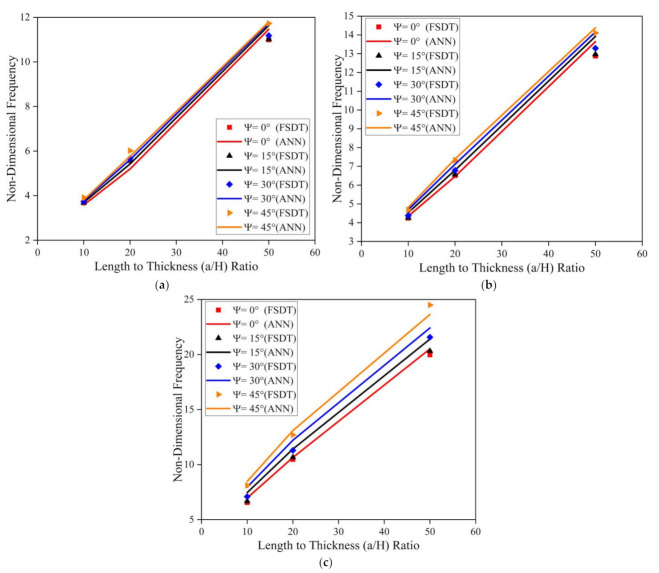
Influence of skew angle on the clamped skew LCS plate operating in an elevated thermal environment (325 K) for varying a/b and a/H ratios (**a**) a/b = 0.5 (**b**) a/b = 1.0 (**c**) a/b = 2.0.

Figure 10 illustrates the effect of skew angle, length to breadth (a/b) ratio, and length to thickness (a/H) ratio on the modal behavior of the simply supported skew LCS plate (with t_c_/t_f_ = 2) in the elevated thermal environment (325 K). Results indicate an upsurge in the frequency value with an increase in a/b and a/H ratios. As the length to thickness (a/H) ratio varies from 10 to 50, the plate transforms from a thick plate to a thin plate condition. As the a/b and a/H ratio increases, the ratio of the magnitude of the stiffness matrix to the mass matrix tends to increase continuously. Many researchers have reported similar observations for various composite structures [19,21,23]. The same trend in variation of natural frequency values for clamped boundary conditions can be observed, as presented in Figure 11.

**Figure 12:**
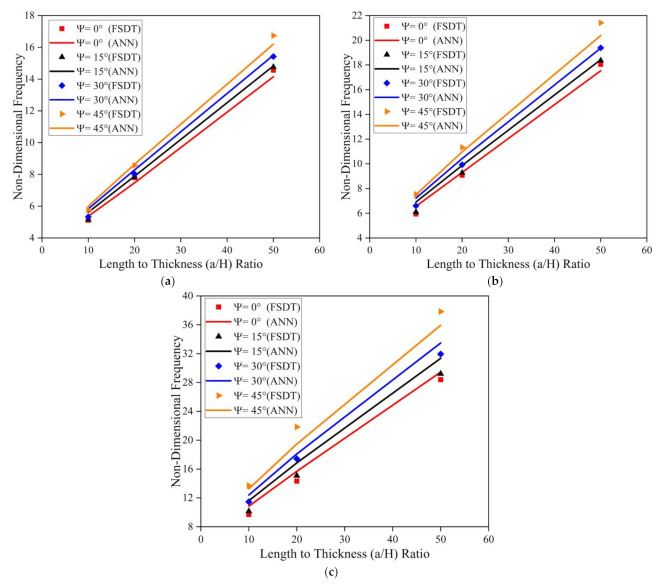
Influence of skew angle on the SSSS skew LCS plate in presence of moisture (0.25%) for varying a/b and a/H ratios (**a**) a/b = 0.5 (**b**) a/b = 1.0 (**c**) a/b = 2.0.

**Figure 13:**
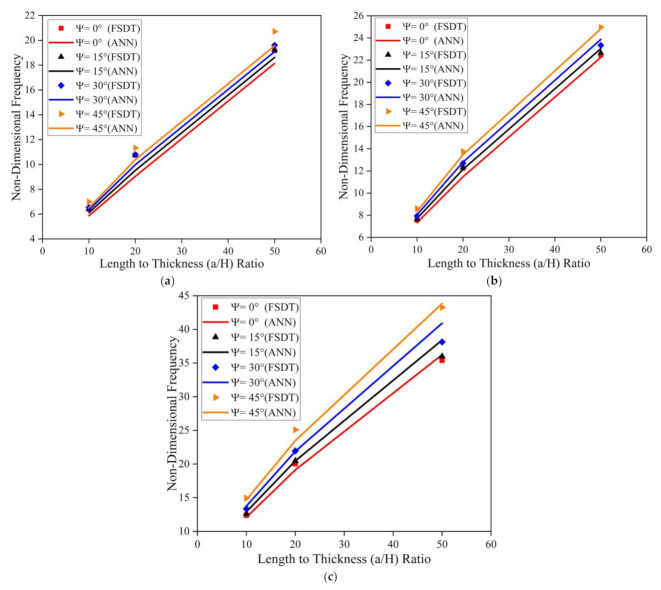
Influence of skew angle on the CCCC skew LCS plate in the presence of moisture (0.25%) for varying a/b and a/H ratios (**a**) a / b = 0.5 (**b**) a / b = 1.0 (**c**) a / b = 2.0.

Simulations were performed to understand the effect of skew angle and the aspect (a/b and a/H) ratios on the fundamental frequency of the SSSS and CCCC skew LCS plates in the presence of moisture (0.25%). From the results presented in Figure 12 and Figure 13, it can be observed that the non-dimensional frequency of the system increases with an increase in the a/b ratio, a/H ratio, and skew angle in the presence of a moisture environment. From Figure 10, Figure 11, Figure 12 and Figure 13, it is clear that the results predicted by the ANN model to understand the effect of the geometrical parameters of the LCS plate such as the a/b ratio, a/H ratio, and skew angle operating in a hygrothermal environment are of good accuracy and are in line with the numerical results.

**Figure 14:**
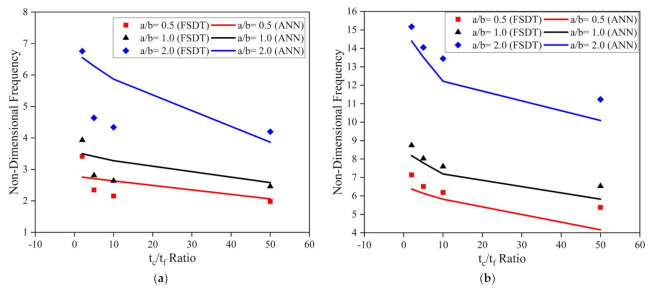
Influence of the t_c_/t_f_ ratio on the clamped LCS plate in the presence of (**a**) temperature (400 K) and (**b**) moisture (1.0%) for varying a/b ratios.

Further investigations were performed to comprehend the influence of the tc/tf ratio on the modal behavior of the LCS plate operating in elevated thermal and moisture environments. The a/H ratio and skew angles were considered as 10 and 45°, respectively, for the analysis. The non-dimensional fundamental frequency values obtained for the clamped LCS plate operating at 400 K for various a/b ratios and the t_c_/t_f_ ratio are presented in Figure 14a. From the results, it is evident that the natural frequency considerably decreases with an increase in the t_c_/t_f_ ratio at an elevated thermal environment. Previously, similar observations were reported by many researchers in ambient and elevated thermal environments [2,39,40]. The clamped LCS plates were also investigated in moisture environments to understand the impact of the t_c_/t_f_ ratio on the natural frequency of the system. The results obtained for the LCS plate operating in a 1% moisture concentration environment are plotted in Figure 14b. From the results, it is evident that the frequency of the system decreases with an increase in moisture concentration value. It can also be observed that for both thermal and moisture cases, the influence of the t_c_/t_f_ ratio is more prominent for higher values of a/b ratios.

**Figure 15:**
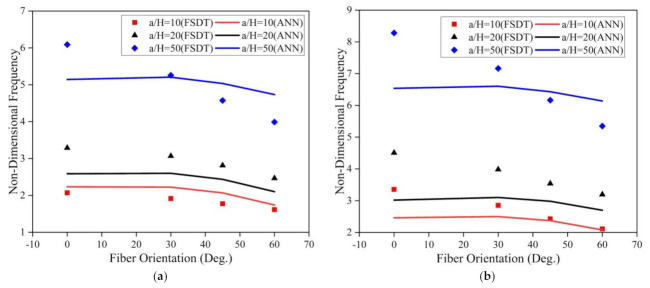
Influence of the face sheet fiber orientation angle on the skew LCS plate in the presence of an elevated thermal environment (375 K) for varying a/H ratios operating under (**a**) SSSS and (**b**) CCCC boundary conditions.

The investigation was further extended to understand the effect of the fiber orientation of the face sheet of the LCS plate on the modal behavior of the system operating in an elevated thermal and moisture environment. The a/b ratio, t_c_/t_f_ ratio, and skew angle were considered as 0.5, 2, and 0°, respectively, for the analysis. The results obtained for the LCS plate operating at a temperature of 375 K for various a/H ratios are presented in Figure 15.

**Figure 16:**
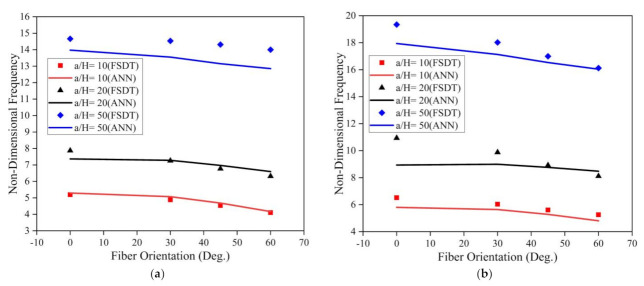
Influence of the face sheet fiber orientation angle on the skew LCS plate in the presence of a moisture environment (0.75 %) for varying a/H ratios operating under (**a**) SSSS and (**b**) CCCC boundary conditions.

From Figure 15, for both SSSS and CCCC conditions, it is evident that the non-dimensional fundamental frequency considerably decreases with an increase in the fiber orientation angle and increases with an increase in the a/H ratio. The investigations were also performed to understand the effect of fiber orientation on the LCS plate operating in a moisture (0.75%) environment. The results obtained are plotted in Figure 16. Results indicate that the fundamental frequency decreases with an increase in the fiber orientation angle from 0° to 60°. From the plots, it is evident that the results predicted by the ANN model follow the same trend as that of the results obtained from the numerical model with good accuracy.

## 5. Conclusions

A methodology was suggested to determine the effect of thermal and moisture environments on the vibration characteristics of skew LCS plates using numerical (FE) and predictive ANN models. The FE model was formulated using FSDT with eight-noded isoparametric elements. The results obtained from the developed FE model were used to train and develop an efficient ANN predictive model to estimate the fundamental frequencies of LCS plates operating in elevated thermal and moisture environments. The ANN predicted results were found to be of good accuracy. The effective utilization of the current model may significantly improve SHM techniques for engineering structures as it can help to detect damage early and suggest subsequent corrective measures. The effect of geometrical parameters such as boundary conditions, stacking sequences, skew angle, and a/b and a/H ratios on the free vibration characteristics were studied. From the simulation results, it is noted that the clamped LCS plates have higher natural frequencies than the simply supported plates. In comparison, the simply supported plates are more susceptible to changing thermal and moisture environments. Further, the non-dimensional frequency increases with an increase in the aspect ratios and skew angles of the LCS plates. Results indicate that the plates with lower skew angles are the least resilient to hygrothermal environments. The natural frequency of the LCS plate noticeably decreases with an increase in the t_c_/t_f_ ratio. It is also noted that non-dimensional fundamental frequency considerably decreases with an increase in the fiber orientation of the face sheet.

## Figures and Tables

**Table 1:** Material Properties [10,38] (Adapted with permission from Elsevier B.V., License Number: 5077641188628 and 5077631407971).

Properties	Units	Graphite-Epoxy [38]	Viscoelastic Core [10]
Elastic Moduli	GPa	Table 2a,b	Figure 2
Density	kg/m^3^	1600	1200
Poisson’s Ratio	m/m	υ12 = 0.3	υ12 = 0.49
Coefficient of moisture expansion	-	β1 = 0 β2 = 0.44	-
Coefficient of thermal expansion	/K	α1 = −0.3 × 10^−6^ α2 = 28.1 × 10^−6^	-

**Table 2:** (**a**) Properties of graphite–epoxy composite at different moisture concentrations G_23_ = 0.5G_12_ [38] (Adapted with permission from Elsevier B.V., License Number: 5077641188628). (**b**) Properties of graphite–epoxy composite at different temperatures G_23_ = 0.5G_12_ [38].

**(a)**					
**Elastic Moduli (GPa)**	**0.00**	**0.25**	**0.50**	**0.75**	**1.00**
E1	130	130	130	130	130
E2	9.50	9.25	9.00	8.75	8.50
G12 = G13	6.0	6.0	6.0	6.0	6.0
**(b)**					
**Elastic Moduli (GPa)**	**300**	**325**	**350**	**375**	**400**
E1	130	130	130	130	130
E2	9.50	8.50	8.00	7.50	7.00
G12 = G13	6.0	6.0	5.5	5.0	4.75

**Table 3:** Influence of hygrothermal environment on the non-dimensional frequency of the clamped skew composite plate.

Skew Angle	Mesh Size	Temperature (K)				Moisture Concentration (%)			
		300	325	350	375	0.00	0.25	0.50	0.75
0°	4 × 4	34.2394	34.2046	34.1697	34.1350	34.2394	34.1720	34.1048	34.0377
	8 × 8	35.6470	35.6113	35.5756	35.5399	35.6470	35.5775	35.5080	35.4386
	12 × 12	35.8753	35.8404	35.8054	35.7705	35.8753	35.8066	35.7381	35.6696
	Ref. [39]	35.8753	35.8404	35.8054	35.7705	35.8753	35.8066	35.7381	35.6696
15°	4 × 4	34.6184	34.5807	34.5431	34.5055	34.6184	34.5470	34.4758	34.4047
	8 × 8	36.0444	36.0060	35.9675	35.9292	36.0444	35.9710	35.8977	35.8246
	12 × 12	36.2630	36.2253	36.1875	36.1498	36.2630	36.1904	36.1179	36.0455
	Ref. [39]	36.2630	36.2253	36.1875	36.1498	36.2630	36.1904	36.1179	36.0455
30°	4 × 4	35.9073	35.8608	35.8143	35.7679	35.9073	35.8232	35.7392	35.6555
	8 × 8	37.3947	37.3477	37.3008	37.2540	37.3947	37.3092	37.2240	37.1390
	12 × 12	37.5810	37.5345	37.4880	37.4415	37.5810	37.4960	37.4111	37.3265
	Ref. [39]	37.5810	37.5345	37.4880	37.4415	37.5810	37.4960	37.4111	37.3265
45°	4 × 4	38.7608	38.6980	38.6352	38.5725	38.7608	38.6532	38.5459	38.4389
	8 × 8	40.3527	40.2904	40.2282	40.1661	40.3527	40.2455	40.1385	40.0319
	12 × 12	40.4746	40.4122	40.3498	40.2875	40.4746	40.3669	40.2594	40.1523
	Ref. [39]	40.4746	40.4122	40.3498	40.2875	40.4746	40.3669	40.2594	40.1523
60°	4 × 4	45.2802	45.1912	45.1024	45.0137	45.2802	45.1346	44.9893	44.8446
	8 × 8	46.9400	46.8524	46.7650	46.6778	46.9400	46.7961	46.6526	46.5097
	12 × 12	46.9691	46.8804	46.7920	46.7037	46.9691	46.8233	46.6781	46.5335
	Ref. [39]	46.9691	46.8804	46.7920	46.7037	46.9691	46.8233	46.6781	46.5335

**Table 4:** Effect of skew angle on the fundamental frequency (Hz) of the LCS plate.

Lamination Scheme	Source	Skew Angles		
		0°	15°	30°
90°/0°/C/0°/90°	FSDT [41]	172.7237	184.5342	225.9660
	HSDT [41]	158.0954	167.8775	201.7029
	Spline finite strip method [40]	159.30	-	-
	Present	157.20	165.10	190.85
0°/90°/C/0°/90°	FSDT [41]	166.3086	177.6942	217.7630
	HSDT [41]	152.2992	161.7182	194.3770
	Spline finite strip method [40]	152.58	-	-
	Present	150.78	158.48	183.61
0°/90°/C/90°/0°	FSDT [41]	159.8275	170.7568	209.3430
	HSDT [41]	146.5089	155.5495	186.9801
	Spline finite strip method [40]	145.99	-	-
	Present	144.40	151.88	176.31

**Table 5:** Parameters considered for the simulations.

Parameters	Range
Temperature (K)	300, 325, 350, 375, 400
Moisture (%)	0, 0.25, 0.5, 0.75, 1
Boundary conditions	SSSS, CCCC
Length to breadth (a/b) ratio	0.5, 1, 2
Length to thickness (a/H) ratio	10, 20, 50
Core thickness to thickness of face sheet (t_c_/t_f_) ratio	2, 5, 10, 50
Fiber orientation of composite face sheets	0°, 30°, 45°, 60°
Skew angle	0°, 15°, 30°, 45°

## Data Availability

Data sharing not applicable.

## Appendix A

(A1) $$\{\varepsilon_{st}\}={\left[\begin{array}{ccc} \frac{\partial u_{0}}{\partial x} & \frac{\partial v_{0}}{\partial y} & \frac{\partial u_{0}}{\partial y}+\frac{\partial v_{0}}{\partial x} \end{array}\right]}^{T};\{\varepsilon_{st}\}={\left[\begin{array}{cc} \frac{\partial w_{0}}{\partial x} & \frac{\partial w_{0}}{\partial y} \end{array}\right]}^{T}$$

(A2) $$\{\varepsilon_{br}\}={\left[\begin{array}{ccccccccc} \frac{\partial \alpha_{x}}{\partial x} & \frac{\partial \alpha_{y}}{\partial y} & \frac{\partial \alpha_{y}}{\partial x}+\frac{\partial \alpha_{x}}{\partial y} & \frac{\partial \beta_{x}}{\partial x} & \frac{\partial \beta_{y}}{\partial y} & \frac{\partial \beta_{y}}{\partial x}+\frac{\partial \beta_{x}}{\partial y} & \frac{\partial \varphi_{x}}{\partial x} & \frac{\partial \varphi_{y}}{\partial y} & \frac{\partial \varphi_{y}}{\partial x}+\frac{\partial \varphi_{x}}{\partial y} \end{array}\right]}^{T}$$

(A3) $$\{\varepsilon_{sr}\}={\left[\begin{array}{cccccc} \alpha_{x} & \alpha_{y} & \beta_{x} & \beta_{y} & \varphi_{x} & \varphi_{y} \end{array}\right]}^{T}$$

(A4) $$[Z_{1}]=\left[\begin{array}{ccccccccc} -h_{c} & 0 & 0 & z+h_{c} & 0 & 0 & 0 & 0 & 0\\ 0 & -h_{c} & 0 & 0 & z+h_{c} & 0 & 0 & 0 & 0\\ 0 & 0 & -h_{c} & 0 & 0 & z+h_{c} & 0 & 0 & 0 \end{array}\right]$$

(A5) $$[Z_{2}]=\left[\begin{array}{ccccccccc} z & 0 & 0 & 0 & 0 & 0 & 0 & 0 & 0\\ 0 & z & 0 & 0 & 0 & 0 & 0 & 0 & 0\\ 0 & 0 & z & 0 & 0 & 0 & 0 & 0 & 0 \end{array}\right]$$

(A6) $$[Z_{3}]=\left[\begin{array}{ccccccccc} h_{c} & 0 & 0 & 0 & 0 & 0 & z-h_{c} & 0 & 0\\ 0 & h_{c} & 0 & 0 & 0 & 0 & 0 & z-h_{c} & 0\\ 0 & 0 & h_{c} & 0 & 0 & 0 & 0 & 0 & z-h_{c} \end{array}\right]$$

(A7) $$[Z_{4}]=\left[\begin{array}{cccccc} 0 & 0 & 1 & 0 & 0 & 0\\ 0 & 0 & 0 & 1 & 0 & 0 \end{array}\right];[Z_{5}]=\left[\begin{array}{cccccc} 1 & 0 & 0 & 0 & 0 & 0\\ 0 & 1 & 0 & 0 & 0 & 0 \end{array}\right]$$

(A8) $$[Z_{6}]=\left[\begin{array}{cccccc} 0 & 0 & 0 & 0 & 1 & 0\\ 0 & 0 & 0 & 0 & 0 & 1 \end{array}\right]$$

## Appendix B

(A9) $$[B_{tbi}]=\left[\begin{array}{ccc} \frac{\partial n_{i}}{\partial x} & 0 & 0\\ 0 & \frac{\partial n_{i}}{\partial y} & 0\\ \frac{\partial n_{i}}{\partial y} & \frac{\partial n_{i}}{\partial x} & 0 \end{array}\right];[B_{tsi}]=\left[\begin{array}{ccc} 0 & 0 & \frac{\partial n_{i}}{\partial x}\\ 0 & 0 & \frac{\partial n_{i}}{\partial y} \end{array}\right];[B_{rsi}]=\left[\begin{array}{cccccc} 1 & 0 & 0 & 0 & 0 & 0\\ 0 & 1 & 0 & 0 & 0 & 0\\ 0 & 0 & 1 & 0 & 0 & 0\\ 0 & 0 & 0 & 1 & 0 & 0\\ 0 & 0 & 0 & 0 & 1 & 0\\ 0 & 0 & 0 & 0 & 0 & 1 \end{array}\right]$$

(A10) $$[B_{rbi}]={\left[\begin{array}{ccc} B_{rbii} & O_{rbii} & O_{rbii}\\ O_{rbii} & B_{rbii} & O_{rbii}\\ O_{rbii} & O_{rbii} & B_{rbii} \end{array}\right]}^{T};\left[B_{rbii}\right]=\left[\begin{array}{ccc} \frac{\partial n_{i}}{\partial x} & 0 & \frac{\partial n_{i}}{\partial y}\\ 0 & \frac{\partial n_{i}}{\partial y} & \frac{\partial n_{i}}{\partial x} \end{array}\right]$$

## Appendix C

(A11) $$\left[K_{11b}^{e}\right]=\int_{A_{e}}{\left[B_{tb}\right]}^{T}\left(\left[D_{11b}^{bot}\right]+\left[D_{11b}^{core}\right]+\left[D_{11b}^{top}\right]\right)\left[B_{tb}\right]dA^{e}$$

(A12) $$\left[K_{12b}^{e}\right]=\int_{A_{e}}{\left[B_{tb}\right]}^{T}\left(\left[D_{12b}^{bot}\right]\left[B_{rb}\right]+\left[D_{12b}^{core}\right]\left[B_{rb}\right]+\left[D_{12b}^{top}\right]\left[B_{rb}\right]\right)dA^{e}$$

(A13) $$\left[K_{22b}^{e}\right]=\int_{A_{e}}\left({\left[B_{rb}\right]}^{T}\left[D_{22b}^{bot}\right]\left[B_{rb}\right]+{\left[B_{rb}\right]}^{T}\left[D_{22b}^{core}\right]\left[B_{rb}\right]+{\left[B_{rb}\right]}^{T}\left[D_{22b}^{top}\right]\left[B_{rb}\right]\right)dA^{e}$$

(A14) $$\left[K_{11s}^{e}\right]=\int_{A_{e}}{\left[B_{ts}\right]}^{T}\left(\left[D_{11s}^{bot}\right]+\left[D_{11s}^{core}\right]+\left[D_{11s}^{top}\right]\right)\left[B_{ts}\right]dA^{e}$$

(A15) $$\left[K_{12s}^{e}\right]=\int_{A_{e}}{\left[B_{ts}\right]}^{T}\left(\left[D_{12s}^{bot}\right]\left[B_{rs}\right]+\left[D_{12s}^{core}\right]\left[B_{rs}\right]+\left[D_{12s}^{top}\right]\left[B_{rs}\right]\right)dA^{e}$$

(A16) $$\left[K_{22s}^{e}\right]=\int_{A_{e}}\left({\left[B_{rs}\right]}^{T}\left[D_{22s}^{bot}\right]\left[B_{rs}\right]+{\left[B_{rs}\right]}^{T}\left[D_{22s}^{core}\right]\left[B_{rs}\right]+{\left[B_{rs}\right]}^{T}\left[D_{22s}^{top}\right]\left[B_{rs}\right]\right)dA^{e}$$

(A17) $$\left[D_{11b}^{bot}\right]=\left[C_{b}^{bot}\right]h_{b};\left[D_{11b}^{core}\right]=2\left[C_{b}^{core}\right]h_{c};\left[D_{11b}^{top}\right]=\left[C_{b}^{top}\right]h_{t}$$

(A18) $$\left[D_{11s}^{bot}\right]=\left[C_{s}^{bot}\right]h_{b};\left[D_{11s}^{core}\right]=2\left[C_{s}^{core}\right]h_{c};\left[D_{ts}^{top}\right]=\left[C_{s}^{top}\right]h_{t}$$

(A19) $$\left[D_{12b}^{bot}\right]=\int_{h_{1}}^{h_{2}}\left[C_{b}^{bot}\right]\left[z_{1}\right]dz;\left[D_{12b}^{core}\right]=\int_{h_{2}}^{h_{3}}\left[C_{b}^{core}\right]\left[z_{2}\right]dz;\left[D_{12b}^{top}\right]=\int_{h_{2}}^{h_{3}}\left[C_{b}^{top}\right]\left[z_{3}\right]dz$$

(A20) $$\left[D_{12s}^{bot}\right]=\int_{h_{1}}^{h_{2}}\left[C_{s}^{bot}\right]\left[z_{4}\right]dz;\left[D_{12s}^{core}\right]=\int_{h_{2}}^{h_{3}}\left[C_{s}^{core}\right]\left[z_{5}\right]dz;\left[D_{12s}^{top}\right]=\int_{h_{2}}^{h_{3}}\left[C_{s}^{top}\right]\left[z_{6}\right]dz$$

(A21) $$\left[D_{22b}^{bot}\right]=\int_{h_{1}}^{h_{2}}{\left[z_{1}\right]}^{T}\left[C_{b}^{bot}\right]\left[z_{1}\right]dz;\left[D_{22b}^{core}\right]=\int_{h_{2}}^{h_{3}}{\left[z_{2}\right]}^{T}\left[C_{b}^{core}\right]\left[z_{2}\right]dz$$

(A22) $$\left[D_{22b}^{top}\right]=\int_{h_{2}}^{h_{3}}{\left[z_{3}\right]}^{T}\left[C_{b}^{top}\right]\left[z_{3}\right]dz;\left[D_{22s}^{bot}\right]=\int_{h_{1}}^{h_{2}}{\left[z_{4}\right]}^{T}\left[C_{s}^{bot}\right]\left[z_{4}\right]dz$$

(A23) $$\left[D_{22s}^{core}\right]=\int_{h_{2}}^{h_{3}}{\left[z_{5}\right]}^{T}\left[C_{s}^{core}\right]\left[z_{5}\right]dz;\left[D_{22s}^{top}\right]=\int_{h_{2}}^{h_{3}}{\left[z_{6}\right]}^{T}\left[C_{s}^{top}\right]\left[z_{6}\right]dz$$

## Appendix D

(A24) $$[S]=\left[\begin{array}{cccc} S_{a} & & & Sym\\ S_{b} & S_{e} & & \\ S_{c} & S_{f} & S_{h} & \\ S_{d} & S_{g} & S_{i} & S_{j} \end{array}\right];[S_{a}]=\left[\begin{array}{cccccc} N_{x} & N_{xy} & 0 & 0 & 0 & 0\\ N_{xy} & N_{y} & 0 & 0 & 0 & 0\\ 0 & 0 & N_{x} & N_{xy} & 0 & 0\\ 0 & 0 & N_{xy} & N_{y} & 0 & 0\\ 0 & 0 & 0 & 0 & N_{x} & N_{xy}\\ 0 & 0 & 0 & 0 & N_{xy} & N_{y} \end{array}\right]$$

(A25) $$[S_{b}]=\left[\begin{array}{cccccc} 0 & 0 & -M_{x} & -M_{xy} & 0 & 0\\ 0 & 0 & -M_{xy} & -M_{y} & 0 & 0\\ M_{x} & M_{xy} & 0 & 0 & 0 & 0\\ M_{xy} & M_{y} & 0 & 0 & 0 & 0\\ 0 & 0 & -M_{x} & -M_{xy} & 0 & 0\\ 0 & 0 & -M_{xy} & -M_{y} & 0 & 0 \end{array}\right]$$

(A26) [Se]=[Sh]=Nxxh212Nxyh2120000Nxyh212Nyyh212000000Nxxh212Nxyh2120000Nxyh212Nyyh212000000Nxxh212Nxyh2120000Nxyh212Nyyh212

(A27) $$[S_{c}]=\left[\begin{array}{cccccc} M_{x} & M_{xy} & 0 & 0 & 0 & 0\\ M_{xy} & M_{y} & 0 & 0 & 0 & 0\\ 0 & 0 & -M_{x} & -M_{xy} & 0 & 0\\ 0 & 0 & -M_{xy} & -M_{y} & 0 & 0\\ M_{x} & M_{xy} & 0 & 0 & 0 & 0\\ M_{xy} & M_{y} & 0 & 0 & 0 & 0 \end{array}\right];[S_{d}]=\left[\begin{array}{cccccc} 0 & 0 & -Q_{x} & -Q_{y} & 0 & 0\\ Q_{x} & Q_{y} & 0 & 0 & 0 & 0\\ 0 & 0 & -Q_{x} & -Q_{y} & 0 & 0\\ Q_{x} & Q_{y} & 0 & 0 & 0 & 0\\ 0 & 0 & -Q_{x} & -Q_{y} & 0 & 0\\ Q_{x} & Q_{y} & 0 & 0 & 0 & 0 \end{array}\right]$$

where,

N_x_, N_y_, N_xy_: In-plane initial internal force resultants per unit length
M_x_, M_y_, M_xy_: Initial internal moment resultants per unit length
Q_x_, Q_y_: Initial transverse shear resultants.

## Appendix E

(A28) $$w_{1}=\left[\begin{array}{cccccccc} 3.7524 & 0.4466 & 5.0637 & 1.7067 & -0.3174 & -2.2000 & 0.0471 & -0.0981\\ 2.0649 & -0.1999 & 2.7643 & -0.2413 & -1.7082 & -0.0936 & -0.0751 & -0.0703\\ 3.6450 & 0.4307 & 4.9177 & 1.6818 & -0.3057 & -4.0082 & 0.0472 & -0.0959\\ -3.4439 & -0.0825 & -4.6190 & 0.2096 & 0.4477 & -0.9254 & 0.0578 & 0.0729\\ 0.4032 & 0.0318 & 0.5317 & 0.3477 & 0.1834 & -0.3018 & 0.2158 & 0.1567\\ 1.6700 & -0.1419 & 2.2369 & -0.1925 & -0.7835 & -0.1024 & -0.0429 & -0.0466\\ -0.8854 & 0.0038 & -1.2460 & 0.2094 & 0.1925 & -0.5381 & 0.6624 & 0.3592\\ -3.3518 & -0.1604 & -4.5060 & 0.1849 & 0.6400 & -1.7129 & 0.0502 & 0.0615 \end{array}\right]$$

(A29) $$b_{1}={\left[\begin{array}{cccccccc} 4.8158 & 2.8938 & 6.6011 & -3.3592 & -2.2787 & 2.2545 & -0.7854 & -4.1450 \end{array}\right]}^{T}$$

(A30) $$w_{2}=\left[\begin{array}{cccccccc} -6.4103 & 3.2353 & 6.5786 & 4.1011 & 2.8221 & -8.2553 & -0.0988 & -2.1999 \end{array}\right]$$

(A31) $$b_{2}=8.4861$$

where

w_1_ and b_1_ are weight and bias of hidden layer
w_2_ and b_2_ are weight and bias of hidden layer
